# Predict potential miRNA-disease associations based on bounded nuclear norm regularization

**DOI:** 10.3389/fgene.2022.978975

**Published:** 2022-08-22

**Authors:** Yidong Rao, Minzhu Xie, Hao Wang

**Affiliations:** College of Information Science and Engineering, Hunan Normal University, Changsha, China

**Keywords:** miRNA, disease, miRNA-disease associations, bounded nuclear norm regularization, matrix completion

## Abstract

Increasing evidences show that the abnormal microRNA (miRNA) expression is related to a variety of complex human diseases. However, the current biological experiments to determine miRNA-disease associations are time consuming and expensive. Therefore, computational models to predict potential miRNA-disease associations are in urgent need. Though many miRNA-disease association prediction methods have been proposed, there is still a room to improve the prediction accuracy. In this paper, we propose a matrix completion model with bounded nuclear norm regularization to predict potential miRNA-disease associations, which is called BNNRMDA. BNNRMDA at first constructs a heterogeneous miRNA-disease network integrating the information of miRNA self-similarity, disease self-similarity, and the known miRNA-disease associations, which is represented by an adjacent matrix. Then, it models the miRNA-disease prediction as a relaxed matrix completion with error tolerance, value boundary and nuclear norm minimization. Finally it implements the alternating direction method to solve the matrix completion problem. BNNRMDA makes full use of available information of miRNAs and diseases, and can deals with the data containing noise. Compared with four state-of-the-art methods, the experimental results show BNNRMDA achieved the best performance in five-fold cross-validation and leave-one-out cross-validation. The case studies on two complex human diseases showed that 47 of the top 50 prediction results of BNNRMDA have been verified in the latest HMDD database.

## 1 Introduction

MicroRNA (miRNA) is a non-coding single-stranded RNA molecule of about 22 nt in length, which have been proved involved in gene regulation by binding to 3’ UTRs of the target mRNAs. It plays a critical role in human cell differentiation, growth, and disease development. Accumulating evidence has shown that miRNAs are closely related to complex human diseases (Liu et al. (2010); Chen et al. (2019); Feng et al. (2012); Zhang et al. (2013)), and discovering miRNA-disease associations is of great significance for the prevention, diagnosis and treatment of human complex diseases. Recently, many miRNA-disease associations have been confirmed and collected in different databases. For example, the HMDD v3.2 database (http://www.cuilab.cn/hmdd) contains 32281 confirmed associations between 850 diseases and 1102 miRNAs (Huang et al. (2019)). However, it is time-consuming and labor intensive for current biological experiments to determine miRNA-disease associations. Therefore, effective computational prediction models are in urgent need.

As so far, a number of computational miRNA-disease associations prediction models have been proposed [Chen et al. (2019); Chen and Zhang (2013); Jiang et al. (2013); Zeng et al. (2016b,a)], and all the models are based on the known miRNA-disease associations to predict the potential associations. For example, based on the known miRNA-disease associations and a miRNA-miRNA functional similarity network, Chen et al. [Chen X. et al. (2012)] developed a method RWRMDA, which used a global network similarity measurement and random walk with restart to predict potential miRNA-disease associations. Based on a miRNA-disease bilayer network constructed according to the above information, Xuan et al. [Xuan et al. (2015)] presented a method using random walk with restart to infer potential associations between miRNAs and diseases. By including an extra disease similarity network, Liao et al. [Liao et al. (2015)] proposed a diffusion-based method NDBM, which also used a global network similarity to predict miRNA-disease associations. Furthermore, Chen et al. (Chen et al. (2016)) integrated the information of miRNA functional similarity, disease semantic similarity, Gaussian interaction profile kernel similarity and the known miRNA-disease associations to build a heterogeneous network, and proposed a new prediction method HGIMDA. You et al. [You et al. (2017)] proposed a path-based prediction model PBMDA, which constructed a similar heterogeneous network and used a depth-first search algorithm to predict potential associations. Based on the heterogeneous network, Chen et al. [Chen et al. (2018c)] proposed a method BNPMDA, which adopted a bipartite network recommendation algorithm to infer potential associations between miRNAs and diseases.

Recently, machine learning methods have been applied to miRNA-disease potential association prediction [Jiang et al. (2013); Chen and Yan (2014); Chen et al. (2018a); Zheng et al. (2019); Zeng et al. (2019); Liang et al. (2019); Li et al. (2020); Zhou et al. (2021)]. For example, Jiang et al. [Jiang et al. (2013)] used support vector machine (SVM) to predict miRNA-disease interaction. Chen et al. [Chen et al. (2018a)] employed a random forest algorithm to predict miRNA-disease associations and proposed a prediction model RFMDA. RFMDA can effectively distinguish related miRNA-disease pairs from unrelated miRNA-disease pairs. Zheng et al. [Zheng et al. (2019)] presented a prediction model MLMDA. MLMDA first used a deep auto-encoder neural network to extract features from the information of disease semantic similarity, Gaussian interaction profile kernel similarity, miRNA functional similarity and miRNA sequences, and adopted a random forest classifier to predict potential associations between miRNAs and diseases based on the extracted features. Liang et al. [Liang et al. (2019)] proposed a method AMVML to infer disease-related miRNAs based on adaptive multi-view multi-label learning. Li et al. [Li et al. (2020)] proposed a miRNA-disease association prediction model NIMCGCN. NIMCGCN used graph convolutional networks to obtain the features of miRNA and disease, and then adopted a neural inductive matrix completion model to infer a new association matrix. Based on graph embedding and multiple meta-paths fusion, Zhang et al. [Zhang et al. (2020)] proposed a model M2GMDA to predict miRNA-disease associations. Based on a heterogeneous network integrating various known associations between miRNA, disease, protein, long non-coding RNA (lncRNA) and drugs, Li et al. [Li H. Y. et al. (2021)] proposed a miRNA-disease association prediction model DF-MDA. DF-MDA adopted a diffusion-based machine-learning method to extract features from the network, and a random forest classifier to predict miRNA-disease associations. Besides, other techniques such as structural deep network embedding [Gong et al. (2019)] and matrix decomposition [Chen et al. (2021); Li L. et al. (2021)] are also used in miRNA-disease association prediction.

To further improve the performance of miRNA-disease association prediction, we propose a novel Bounded Nuclear Norm Regularization based miRNA-disease association prediction model BNNRMDA. At first, BNNRMDA integrates the information of the disease semantic similarity, the miRNA functional similarity, the Gaussian interaction profile kernel similarity and the experimentally verified miRNA-disease associations to construct a heterogeneous miRNA-disease network. Since the number of verified miRNA-disease associations is very small than the total miRNA-disease pairs, the adjacent matrix of the network is sparse, BNNRMDA uses a bounded nuclear norm regularization method to complete the sparse matrix, and the element value of the completed matrix indicates the likelihood that the corresponding miRNA and disease are related. The experiments of leave-one-out cross-validation and five-fold cross-validation in a benchmark dataset showed that BNNRMDA is effective to predict potential miRNA-disease associations. In addition, case studies of colon neoplasms and lung neoplasms showed that the accuracy of BNNRMDA reached 94%.

## 2 Methods

**FIGURE 1:**
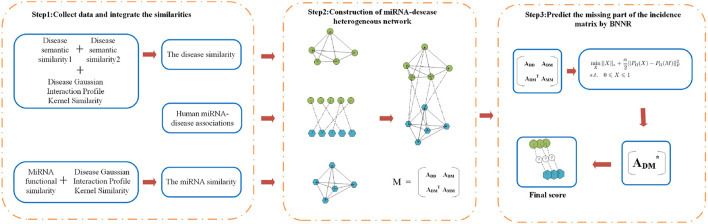
Flowchart of BNNRMDA. The first step collects the information of known miRNA–disease associations, the disease similarity and the miRNA similarity. The second step constructs a heterogeneous miRNA-disease network. The third step uses a matrix completion method BNNR (bounded nuclear norm regularization) to calculate a score for the miRNA-disease pairs with unknown relationship.

Figure 1 gives a flowchart of BNNRMDA. The process of BNNRMDA consists of 3 steps. The first step collects the information of known miRNA–disease associations, the disease similarity and the miRNA similarity. The second step constructs a heterogeneous miRNA-disease network and obtain the corresponding adjacent matrix **M**. The third step uses a matrix completion method to complete **M**, and predicts potential miRNA-disease associations based on the completed matrix.

### 2.1 Data and similarity measures

#### 2.1.1 Validated human miRNA-disease associations

As most of similar works, the HMDD v2.0 database [Li et al. (2014)] was used as the benchmark dataset, which contains 5430 verified associations between 495 miRNAs and 383 diseases. For convenience, we used a *n*_*d*_ × *n*_*m*_ binary matrix *A*_DM_ to store the validated associations from the database with *n*_*d*_ = 383 and *n*_*m*_ = 495. If the *i*th disease has a known association with the *j*th miRNA, then the element $A_{{\text{DM}}_{i,j}}$ is set to 1, otherwise it is 0.

#### 2.1.2 MiRNA functional similarity

Based on the hypothesis that miRNAs with similar functions are more likely to be related to similar diseases, Wang et al. [Wang et al. (2010)] calculated the similarity between miRNAs based on the similarity of their associated disease DAGs. The miRNA functional similarity data was directly downloaded via the link http://www.cuilab.cn/files/images/cuilab/misim.zip provided by Wang et al. [Wang et al. (2010)]. We used a matrix *FS* to represent the data, where the element *FS*_*i*,*j*_ represents the functional similarity score between the *i*th miRNA and the *j*th miRNA.

#### 2.1.3 Disease semantic similarity

We combined two disease semantic similarity measures to calculate the semantic similarity between two diseases. The first was introduced by Wang et al. (Wang et al. (2010)), which is based on the medical subject headings (MeSH) descriptors. The MeSH descriptor of a disease is organized as a hierarchical directed acyclic graph (DAG) with each node is a disease term. For a disease D, let the DAG corresponding to its MeSH descriptor be DAG(*D*) = (*T*(*D*), *E*(*D*)). *T*(*D*) includes the node D and its ancestor nodes (more general disease term), and *E*(*D*) is the set of direct edges representing the parent-child relationship between the disease terms.

The semantic contribution of a disease term *t* to *D* in DAG(*D*) is defined as Eq. 1.

$$C_{1_{D}}\left(t\right)=\left\{\begin{array}{cc} 1\; & \text{ if }t=D\\ \max\left\{\theta C_{1_{D}}\left(t^{\prime }\right)|t^{\prime }\text{ is a child of }t\right\}\; & \text{ if }t\neq D \end{array}\right.$$ (1)

where *θ* is the semantic contribution factor, and is set 0.5 as suggested in Wang et al. (2010).

The semantic value of disease *D* is calculated by Eq. 2, and the Wang’s similarity between diseases *d*_*i*_ and *d*_*j*_ is defined as Eq. 3.

$$V_{1}\left(D\right)=\underset{d\in T\left(D\right)}{\sum }C_{1_{D}}\left(d\right).$$ (2)

$${\text{SS}}_{1}\left(d_{i},d_{j}\right)=\frac{\sum_{t\in T\left(d_{i}\right)\cap T\left(d_{j}\right)}\left(C_{1_{d_{i}}}\left(t\right)+C_{1_{d_{j}}}\left(t\right)\right)}{V_{1}\left(d_{i}\right)+V_{1}\left(d_{j}\right)}.$$ (3)

The second disease similarity measure was introduced by Xuan et al. (Xuan et al. (2013)), and it is defined as Eq. 4.

$${\text{SS}}_{2}\left(d_{i},d_{j}\right)=\frac{2\sum_{t\in T\left(d_{i}\right)\cap T\left(d_{j}\right)}\text{IC}\left(t\right)}{\sum_{t\in T\left(d_{i}\right)}\text{IC}\left(t\right)+\sum_{t\in T\left(d_{j}\right)}\text{IC}\left(t\right)}.$$ (4)

IC(*t*) is the information content of the likelihood of *t* occurring as a node in a disease DAG, and can be calculated as follows.

$$\text{IC}\left(t\right)=-\text{log}\left[\frac{\text{the number of DAGs containing }t}{\text{the total number of DAGs}}\right].$$ (5)

Finally, we average the above two similarity measures of *d*_*i*_ and *d*_*j*_ and obtain the combined disease semantic similarity.

$$\text{SS}\left(d_{i},d_{j}\right)=\frac{{\text{SS}}_{1}\left(d_{i},d_{j}\right)+{\text{SS}}_{2}\left(d_{i},d_{j}\right)}{2}.$$ (6)

#### 2.1.4 Gaussian interaction profile kernel similarity

Based on similar diseases may be related to miRNAs with similar functions, Gaussian interaction profile kernel (GIPK) similarity has been widely used to calculate miRNA similarity and disease similarity. Let *K*(*d*_*i*_) be the vector containing elements at the *i*th row of the binary miRNA-disease association matrix *A*_DM_, and *K*(*m*_*j*_) be the vector containing elements at the *j*th column of *A*_DM_. *K*(*d*_*i*_) and *K*(*m*_*j*_) represent the interaction profiles of disease *d*_*i*_ and miRNA *m*_*j*_ respetively.

The equations to calculate the disease GIPK similarity and the miRNA GIPK similarity are as follows.

$$\text{GKSD}\left(d_{i},d_{j}\right)=\exp\left(-\rho_{d}\|K\left(d_{i}\right)-K\left(d_{j}\right){\|}^{2}\right)$$ (7)

$$\text{GKSM}\left(m_{i},m_{j}\right)=\exp\left(-\rho_{m}\|K\left(m_{i}\right)-K\left(m_{j}\right){\|}^{2}\right)$$ (8)

The coefficients *ρ*_*d*_ and *ρ*_*m*_ are defined in the following equations.

$$\rho_{d}=\frac{n_{d}}{\sum_{i=1}^{n_{d}}\|K\left(d_{i}\right){\|}^{2}}$$ (9)

$$\rho_{m}=\frac{n_{m}}{\sum_{i=1}^{n_{m}}\|K\left(m_{i}\right){\|}^{2}}$$ (10)

### 2.2 Similarity integration and heterogeneous network construction

Since some diseases do not have any MeSH descriptor, we cannot calculate the semantic similarity between these diseases and others. In the case, we use GIPK similarity to replace the semantic similarity. Similarly, when the functional similarity between two miRNAs is missing, the corresponding GIPK similarity is used instead. Finally we obtain a disease similarity matrix A_DD_ and a miRNA similarity matrix A_MM_ as follows.

$${\text{A}}_{{\text{DD}}_{i,j}}=\left\{\begin{array}{cc} \text{SS}\left(d\left(i\right),d\left(j\right)\right)\; & \text{if }\text{SS}\left(d\left(i\right),d\left(j\right)\right)\neq 0,\\ \text{GKSD}\left(d\left(i\right),d\left(j\right)\right)\; & \text{otherwise.} \end{array}\right.$$ (11)

$${\text{A}}_{{\text{MM}}_{i,j}}=\left\{\begin{array}{cc} \text{FS}\left(m\left(i\right),m\left(j\right)\right)\; & \text{if }\text{FS}\left(m\left(i\right),m\left(j\right)\right)\neq 0,\\ \text{GKSM}\left(m\left(i\right),m\left(j\right)\right)\; & \text{otherwise}. \end{array}\right.$$ (12)

We integrate the information of disease similarity, miRNA similarity, the known miRNA-disease associations into a heterogeneous miRNA-disease network. The heterogeneous miRNA-disease network is encoded into a (*n*_*d*_ + *n*_*m*_) × (*n*_*d*_ + *n*_*m*_) matrix M as follows.

$$M=\left[\begin{array}{cc} {\text{A}}_{\text{DD}} & {\text{A}}_{\text{DM}}\\ {{\text{A}}_{\text{DM}}}^{T} & {\text{A}}_{\text{MM}} \end{array}\right].$$

### 2.3 Matrix completion with bounded nuclear norm regularization

Since the verified miRNA-disease associations are much less than the total miRNA-disease pairs, A_DM_ is very sparse (most elements are 0). The miRNA-disease association prediction problem can be model as the matrix completion problem of M. If M_*i*,*j*_ corresponds to a known miRNA-disease association, indicates a miRNA similarity or a disease similarity, it called a known entry. Let Ω = {(*i*, *j*)∣M_*i*,*j*_ is a known entry}. The goal of our miRNA-disease association prediction is to find appropriate values for the unknown entries of M as the final miRNA-disease association prediction scores.

The matrix completion problem of M is generally formulated as find a matrix M* such that the projections of M* and M onto Ω are equal and the rank of M* is minimized, and the formulation is as follows:

$$\begin{array}{c} \underset{{\text{M}}^{*}}{\min}\text{rank}\left(M^{*}\right)\\ s.t.\;\;P_{\Omega }\left(M^{*}\right)=P_{\Omega }\left(M\right), \end{array}$$

where $P_{\Omega }\left(\cdot \right)$ is the projection function such that

$$P_{\Omega }{\left(X\right)}_{ij}=\left\{\begin{array}{cc} X_{ij}, & \left(i,j\right)\in \Omega \\ 0, & \left(i,j\right)\notin \Omega \end{array}.\right.$$

However the rank minimization matrix completion problem is NP-hard. Inspired by Yang et al. (2019), we model the miRNA-disease association prediction as a relaxed matrix completion with error tolerance, value boundary and nuclear norm minimization, which is called the BNNR (bounded nuclear norm regularization) model (Yang et al. (2019)) and is formulated as follows.

$$\begin{array}{c} \underset{X}{\min}\|X{\|}_{*}+\frac{\alpha }{2}\|P_{\Omega }\left(X\right)-P_{\Omega }\left(\text{M}\right){\|}_{F}^{2}\\ s.t.\;0⩽X⩽1, \end{array}$$ (13)

where ‖*X*‖_*_ is the nuclear norm of *X*, i.e. the sum of all singular values of *X*. ‖ ⋅‖_*F*_ is the Frobenius norm and *α* is the parameter that balances the nuclear norm and error term. The BNNR model is a convex optimization problem, and many effective algorithms such as AMM (alternating direction method) (Chen C. H. et al. (2012)) could be used solve it.

To use AMM (Chen C. H. et al. (2012)) to solve the BNNR model, we introduce an auxiliary matrix *H*, and the BNNR model is equivalent to the following model.

$$\begin{array}{c} \underset{X}{\min}\|X{\|}_{*}+\frac{\alpha }{2}\|P_{\Omega }\left(X\right)-P_{\Omega }\left(\text{M}\right){\|}_{F}^{2}\\ s.t.\;\;X=H,\;0⩽H⩽1. \end{array}$$ (14)

Therefore, the extended Lagrange function is:

$$L\left(H,X,Y,\alpha ,\beta \right)=\|X{\|}_{*}+\frac{\alpha }{2}\|P_{\Omega }\left(X\right)-P_{\Omega }\left(M\right){\|}_{F}^{2}+\mathrm{Tr}\left(Y^{T}\left(X-H\right)\right)+\frac{\beta }{2}\|X-H{\|}_{F}^{2},$$ (15)

where *β* > 0 is the penalty parameter and *Y* is the Lagrange multiplier. The model (14) could be solved by an iterative process, whose details could be found in (Yang et al. (2019).

After a series of iterations, a convergent *H* would be finally obtained, which is denoted by H*. Let

$${\text{M}}^{*}=\left[\begin{array}{cc} {\text{A}}_{\text{DD}}^{*} & {\text{A}}_{\text{DM}}^{*}\\ {{\text{A}}_{\text{DM}}^{*}}^{T} & {\text{A}}_{\text{MM}}^{*} \end{array}\right]=H^{*}.$$ (16)

The predicted miRNA-disease associations are found from matrix ${\text{A}}_{\text{DM}}^{*}$ .

## 3 Results

### 3.1 Parameter setting

**FIGURE 2:**
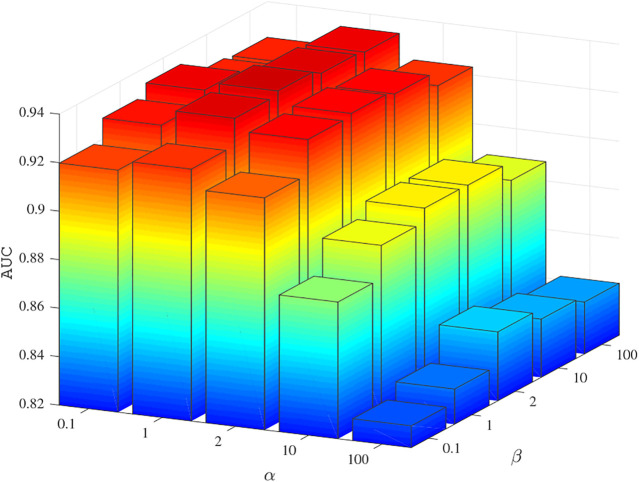
The AUC values using different *α* and *β* values in five fold CV experiments on the training dataset.

The values of parameters *α* and *β* were determined by 5-fold cross-validation experiments on the benchmark dataset. The values were chosen from 0.1, 1, 2, 10, 100, and the AUC results are shown in Figure 2. The experimental results show that when *α* = 1 and *β* = 2, BNNRMDA achieved the best performance. Therefore, in the following experiments, we set *α* = 1 and *β* = 2.

### 3.2 Performance evaluation

We compared BNNRMDA with four state-of-the-art methods IMCMDA (Chen et al. (2018b)), KATZBNRA (Li et al. (2019)), PMFMDA (Xu et al. (2019)) and WBNPMD (Xie et al. (2019)) using global leave-one-out cross-validation (LOOCV) and 5-fold cross-validation (5-fold CV).

**FIGURE 3:**
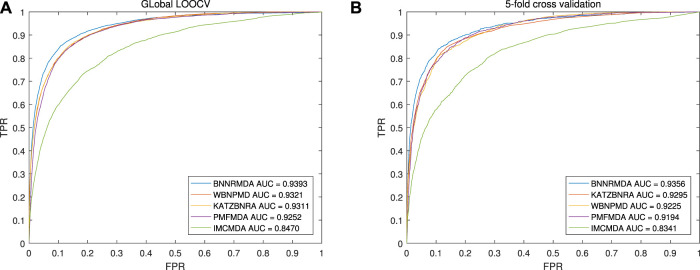
Performance comparisons of BNNRMDA with baseline methods (WBNPMD, KATZBNRA, PMFMDA, IMCMDA) in terms of AUC based on **(A)** the global LOOCV scheme and **(B)** 5-CV scheme.

The benchmark dataset was from the HMDD v2.0 database, which contains 5430 known miRNA-disease associations. Under the global LOOCV framework, each known association is selected out for testing, the others are used as the training set, and all unknown miRNA-disease associations will be used as candidate associations. After BNNRMDA calculates all associated prediction scores, the rank of each test sample will be obtained by comparing with the candidate samples. Higher the rank of the test sample, more effective our model is. We changed the threshold to calculate the true positive rate (TPR) and false positive rate (FPR) and drew the ROC curve. The area under the ROC curve (AUC) was calculated to compare the performance. The higher the AUC value, the better the performance of the model. The experimental results of the global LOOCV is shown in Figure 3 (a). The AUC values of BNNRMDA, IMCMDA, KATZBNRA, PMFMDA, and WBNPMD are 0.9393, 0.8470, 0.9311, 0.9252, 0.9321 respectively.

In the 5-CV framework, all known miRNA-disease association pairs will be randomly divided into five parts; in each experiment, one part is tested, and the other four parts are used as a training set. Similar to LOOCV, the AUC values are used to compare the performance of these models. Figure 3 (b) shows the results of the 5CV experiment, and BNNRMDA achieved the best AUC of 0.9356. The AUC values of IMCMDA, KATZBNRA, PMFMDA, and WBNPMD are 0.8341, 0.9295, 0.9194, 0.9225 respectively.

### 3.3 Case studies

In order to further verify the effect of the BNNRMDA model, we conducted case studies on two human diseases colon neoplasms and lung neoplasms. These diseases pose a great threat to human life. For example, lung neoplasms are one of the common neoplasms in the human body. In recent years, a large number of colon neoplasms cases have died, posing a major threat to human life (DeSantis et al. (2019)). For colon neoplasms, after removing all known related miRNA-disease pairs, we rank the final prediction results of the miRNA related to them. We use two miRNA-disease association databases for verification, namely the dbDEMC (Yang et al. (2017)) database and the HMDD (Huang et al. (2019)) database. As can be clearly seen in Table 1, 47 of the top 50 prediction results have been confirmed to be related in dbDEMC2.0 and HMDD v3.2. Similarly, the results of the top 50 miRNAs predicted for lung neoplasms are shown in Table 2. Among them, 47 of the top 50 can be confirmed in dbDEMC2.0 and HMDD v3.2.

**TABLE 1:** The top 50 potential miRNAs associated with colon neoplasms.

(2 pt) miRNA	Evidence	miRNA	Evidence
hsa-mir-155	dbDEMC;HMDD	hsa-mir-31	dbDEMC;HMDD
hsa-mir-21	dbDEMC;HMDD	hsa-mir-146b	dbDEMC
hsa-mir-146a	dbDEMC;HMDD	hsa-mir-141	dbDEMC;HMDD
hsa-mir-20a	dbDEMC;HMDD	hsa-mir-199a	unconfirmed
hsa-mir-16	dbDEMC	hsa-mir-24	dbDEMC;HMDD
hsa-mir-125b	dbDEMC;HMDD	hsa-let-7a	dbDEMC;HMDD
hsa-mir-15b	dbDEMC;HMDD	hsa-mir-150	dbDEMC;HMDD
hsa-mir-29b	dbDEMC;HMDD	hsa-mir-200b	dbDEMC;HMDD
hsa-mir-143	dbDEMC;HMDD	hsa-mir-7	dbDEMC
hsa-mir-101	HMDD	hsa-mir-9	dbDEMC
hsa-mir-19b	dbDEMC	hsa-mir-148a	dbDEMC;HMDD
hsa-mir-34a	dbDEMC;HMDD	hsa-let-7c	dbDEMC;HMDD
hsa-mir-29a	dbDEMC;HMDD	hsa-mir-221	dbDEMC;HMDD
hsa-mir-106b	dbDEMC;HMDD	hsa-mir-23a	dbDEMC;HMDD
hsa-mir-19a	dbDEMC;HMDD	hsa-mir-107	dbDEMC;HMDD
hsa-mir-196a	dbDEMC;HMDD	hsa-mir-133b	dbDEMC;HMDD
hsa-mir-125a	dbDEMC;HMDD	hsa-mir-34c	unconfirmed
hsa-mir-1	dbDEMC;HMDD	hsa-mir-25	dbDEMC;HMDD
hsa-mir-15a	dbDEMC;HMDD	hsa-mir-30c	dbDEMC;HMDD
hsa-mir-223	dbDEMC;HMDD	hsa-mir-29c	dbDEMC
hsa-mir-214	dbDEMC	hsa-let-7b	dbDEMC;HMDD
hsa-mir-133a	dbDEMC;HMDD5	hsa-mir-26a	unconfirmed
hsa-mir-132	dbDEMC;HMDD	hsa-mir-203	dbDEMC;HMDD
hsa-mir-18a	dbDEMC;HMDD	hsa-let-7i	dbDEMC;HMDD
hsa-mir-92a	dbDEMC;HMDD	hsa-mir-222	dbDEMC;HMDD

**TABLE 2:** The top 50 potential miRNAs associated with lung neoplasms.

(2 pt) miRNA	Evidence	miRNA	Evidence
hsa-mir-106b	dbDEMC	hsa-mir-429	dbDEMC
hsa-mir-20b	dbDEMC	hsa-mir-296	unconfirmed
hsa-mir-130a	dbDEMC;HMDD	hsa-mir-129	dbDEMC;HMDD
hsa-mir-16	dbDEMC;HMDD	hsa-mir-708	dbDEMC
hsa-mir-23b	dbDEMC	hsa-mir-211	dbDEMC
hsa-mir-342	dbDEMC;HMDD	hsa-mir-196b	dbDEMC;HMDD
hsa-mir-15a	dbDEMC;HMDD	hsa-mir-302c	dbDEMC
hsa-mir-378a	unconfirmed	hsa-mir-302b	dbDEMC
hsa-mir-195	dbDEMC;HMDD	hsa-mir-328	dbDEMC;HMDD
hsa-mir-15b	dbDEMC	hsa-mir-99b	dbDEMC
hsa-mir-122	dbDEMC;HMDD	hsa-mir-149	dbDEMC;HMDD
hsa-mir-193b	dbDEMC	hsa-mir-423	HMDD
hsa-mir-424	dbDEMC	hsa-mir-152	dbDEMC;HMDD
hsa-mir-144	dbDEMC;HMDD	hsa-mir-449b	dbDEMC
hsa-mir-92b	dbDEMC	hsa-mir-194	dbDEMC;HMDD
hsa-mir-130b	dbDEMC;HMDD	hsa-mir-208a	HMDD
hsa-mir-204	dbDEMC	hsa-mir-302a	dbDEMC
hsa-mir-451a	dbDEMC;HMDD	hsa-mir-491	dbDEMC
hsa-mir-99a	dbDEMC;HMDD	hsa-mir-452	dbDEMC
hsa-mir-449a	dbDEMC;HMDD	hsa-mir-373	dbDEMC;HMDD
hsa-mir-10a	dbDEMC;HMDD	hsa-mir-625	dbDEMC
hsa-mir-141	dbDEMC;HMDD	hsa-mir-181d	dbDEMC
hsa-mir-139	dbDEMC;HMDD	hsa-mir-367	dbDEMC
hsa-mir-151a	unconfirmed	hsa-mir-520a	dbDEMC
hsa-mir-28	dbDEMC	hsa-mir-520d	dbDEMC

## 4 Conclusion

We proposed a new miRNA-disease association prediction model BNNRMDA. BNNRMDA constructs a miRNA-disease heterogeneous network by integrating miRNA similarity network, disease similarity network and miRNA-disease known association network, and formulates the miRNA-disease association prediction problem as a relaxed matrix completion with error tolerance, value boundary and nuclear norm minimization (BNNR), and at last uses alternating direction method (AMM) to obtain an optimal solution. The global leave-one-out cross-validation experiments and the five-fold cross-validation framework experiments on the benchmark dataset show that BNNRMDA performs better than four state-of-the-art methods. In addition, the case studies on two complex human diseases also illustrate the reliability of BNNRMDA. BNNRMDA can be an effective tool to identify potential miRNA-disease associations. There are some factors that will affect the final prediction results of BNNRMDA. First of all, the materials we used include experimentally verified miRNA-disease associations, miRNA functional similarities, and disease semantic similarities. These data may contain noises and outliers, and appropriate preprocess of the data might enhance the prediction accuracy of BNNRMDA. The choice of parameters *α* and *β* has a certain impact on the prediction performance, and how to choose appropriate parameters based on some statistical characteristics of data is challenging.

## Data Availability

Publicly available datasets were analyzed in this study. This data can be found here: http://www.cuilab.cn/files/images/cuilab/misim.zip.
